# Low Birth Weight Is Strongly Associated with the Risk of Deep Infiltrating Endometriosis: Results of a 743 Case-Control Study

**DOI:** 10.1371/journal.pone.0117387

**Published:** 2015-02-13

**Authors:** Bruno Borghese, Jeanne Sibiude, Pietro Santulli, Marie-Christine Lafay Pillet, Louis Marcellin, Ivo Brosens, Charles Chapron

**Affiliations:** 1 Institut Cochin, Université Paris Descartes, Sorbonne Paris Cité, CNRS UMR 8104, Paris, France; 2 Inserm, U1016, Paris, France; 3 Université Paris Descartes, Sorbonne Paris Cité, Service de Gynécologie Obstétrique 2 et Médecine de la Reproduction, Groupe Hospitalier Cochin, AP-HP, Paris, France; 4 Université Paris Descartes, Sorbonne Paris Cité, Faculté de Médecine, EA 1833, ERTi, Groupe Hospitalier Cochin, AP-HP, Paris, France; 5 Catholic University of Leuven, and Leuven Institute for Fertility and Embryology, Leuven, Belgium; Zhejiang University School of Medicine, CHINA

## Abstract

The influence of intrauterine environment on the risk of endometriosis is still controversial. Whether birth weight modifies the risk of endometriosis in adulthood remains an open question. For this purpose, we designed a case-control study involving 743 women operated on for benign gynecological indications from January 2004 to December 2011. Study group included 368 patients with histologically proven endometriosis: 54 superficial endometriosis (SUP), 79 endometriomas (OMA) and 235 deep infiltrating endometriosis (DIE). Control group included 375 patients without endometriosis as surgically checked. Mean birth weights were compared between patients and controls, according to endometriosis groups and rAFS stages. Mean birth weight was significantly lower for patients with endometriosis as compared to controls (3,119g ± 614 and 3,251g ± 557 respectively; p = 0.002). When compared to controls, patients with DIE had the lowest birth weight with a highly significant difference (3,103g ± 620, p = 0.002). In univariate analysis, patients with low birth weight (LBW), defined as a BW < 2,500g, had a higher risk of endometriosis, especially DIE, as compared to the reference group (OR = 1.5, 95%CI: 1.0-2.3 and OR = 1.7, 95%CI: 1.0-2.7, respectively). Multivariate analysis, adjusted on ethnicity and smoking status, showed the persistence of a significant association between endometriosis and LBW with a slight increase in the magnitude of the association (aOR = 1.7, 95%CI: 1.0-2.6 for endometriosis, aOR = 1.8; 95%CI: 1.1-2.9 for DIE). In conclusion, LBW is independently associated with the risk of endometriosis in our population. Among patients with LBW, the risk is almost two-times higher to develop DIE. This association could reflect common signaling pathways between endometriosis and fetal growth regulation. There is also the possibility of a role played by placental insufficiency on the development of the neonate’s pelvis and the occurrence of neonatal uterine bleeding that could have consequences on the risk of severe endometriosis.

## Introduction

In the past few years, we and others identified some epidemiologic markers associated with a higher risk of endometriosis, including body mass index (BMI) (association between a BMI < 18.5 and deep infiltrating endometriosis (DIE)) [1] and oral contraceptive pill (especially when indicated for NSAID-resistant dysmenorrhea) [2,3], but not smoking [4]. These markers may be useful for two main reasons: (i) they can help to shorten the time required to diagnose and properly handle the disease. This delay is currently around 8 to 10 years, which is far too long, especially for infertile women [5]. Gynecologists and general practitioners should ask about these factors to better identify the patients who are at risk of severe endometriosis [6]; (ii) they provide insight into the disease and contribute to the development of new treatments.

Endometriosis may be influenced by environmental factors, especially during pregnancy [7–9]. Prenatal or perinatal exposure to various external influences could induce endometrial changes in the newborn and promote the development of endometriosis later in life [10]. In children born with low birth weight (LBW) (i.e. weight at term delivery below 2,500g), the risk of metabolic syndrome and other vascular diseases in adulthood is well documented. There is a relationship between LBW and subsequent vascular risk, including metabolic syndrome, cardiovascular disease, type 2-diabetes and obesity in adulthood. The risk of metabolic syndrome is ten-times higher in subjects with a birth weight of less than 2,500g, compared to those with normal birth weight [11]. Young women being born at LBW, irrespective of gestational age, exhibit a reduction in insulin sensitivity and an increased risk of developing clinical and biochemical features of polycystic ovary syndrome [12].

Therefore it seems relevant to study the potential association between birth weight and endometriosis. For this purpose, we designed a case-control study including 368 patients with histologically confirmed endometriosis and 375 healthy controls in whom the disease was excluded by surgical examination. Birth weights were collected from medical charts for each participant.

## Materials and Methods

We conducted a case-control study using data extracted from a prospectively managed database, as previously described [3]. From January 2004 to December 2011, all women younger than 42 years old who were referred to our institution for surgery by operative laparoscopy or laparotomy were asked to take part in the study. Exclusion criteria were the followings: (i) normal intra-uterine pregnancy; (ii) diagnosis of cancer; (iii) incomplete surgical excision of endometriotic lesions; (iv) absence of histologic confirmation of endometriosis; (v) absence of data on birth weight. Indications for surgery, sometimes more than one per patient, were as follows: (i) preoperative assessment of endometriosis by magnetic resonance imaging and/or ultrasound; (ii) pelvic pain, defined as the presence for at least 6 months of dysmenorrhea and/or intermenstrual pelvic pain and/or dyspareunia of moderate to severe intensity; (iii) infertility defined as at least 12 months of unprotected intercourse not resulting in pregnancy; (iv) pelvic mass (benign ovarian cyst, uterine myoma, etc.); (v) others: uterine bleeding, request for tubal ligation, tubal infection, etc. No specific protocols were used for medical management prior to surgery.

Study group included patients with histologically proven endometriosis. Control group included patients without any lesions suggestive of endometriosis as thoroughly checked during the surgical procedure. Based on histological findings, endometriotic lesions were classified into three groups: superficial peritoneal endometriosis (SUP), ovarian endometrioma (OMA) and deeply infiltrating endometriosis (DIE) [3]. DIE was defined as endometriotic lesions infiltrating the *muscularis propria* of utero-sacral ligament, vagina, bladder, intestine or ureter [13]. As these groups are frequently associated with each other [14], patients were arbitrarily classified into the group of the “worst” finding, i.e. from least to worst: SUP, OMA and DIE [4]. The extent of endometriosis was staged during the surgery according to the revised American Fertility Society (rAFS) classification [15]. Total rAFS score and scores for implants and adhesions were assessed according to the same scoring system.

For each patient, data were recorded one month before surgery in face-to-face interviews using a previously published questionnaire [4]. We collected general information such as age, parity, gravidity, body mass index, primary or secondary infertility and its duration, pelvic pain scores and associated symptoms (dysmenorrhea, deep dyspareunia, non-cyclic chronic pelvic pain, gastro-intestinal symptoms and lower urinary tract symptoms) and lifestyle habits [3,4].

Birth weights were collected from maternity records. When the maternity record was not available, the patient was not included in the study. Women were categorized according to birth weight into four groups defined as follows: ≤ 1,500g, 1,501g—2,500g, 2,501g—4,000g and > 4,000g.

All statistical data were collected in a computerized database. Statistical analysis was performed using STATA 11.0 software (Stata Corp., College Station, Texas, USA). The continuous data were presented as mean and mean standard deviation. Student’s t-test and ANOVA were carried out when appropriate. The chi-square or Fischer’s exact tests were used for categorical data. Average birth weights were first compared for all cases and controls. Then, groups of endometriosis (SUP, OMA and DIE) [16] and disease severity (rAFS classification) [15] were compared to controls. We performed an unconditional logistic regression to control for potential confounding factors associated with birth weight and endometriosis risk, such as ethnicity and smoking status. We computed, in the four intervals of weight (≤ 1,500g, 1,501g—2,500g, 2,501g—4,000g and > 4,000g), the crude and multivariate adjusted odds ratios (aOR) and the corresponding 95% confidence intervals (CI) for the risk of endometriosis, SUP, OMA and DIE compared to the control group. Multivariate models were adjusted on ethnicity and smoking status, assuming ethnicity could be linked both to birth weight and endometriosis, and smoking status could be linked to smoking status of the parents, and thus to birth weight. Other variables were not considered as potential confounders. For the analyses, women with birth weight interval between 2,501g and 4,000g served as the reference population.

A two-sided p-value of 0.05 was considered as statistically significant. The study was approved by the local institutional review board (IRB approval number 05–2006 given by the ‘Comité de Protection des Personnes et des Biens dans la Recherche Biomédicale’ of Paris Cochin) and written informed consent was obtained from all the participating subjects.

## Results

**Fig 1:**
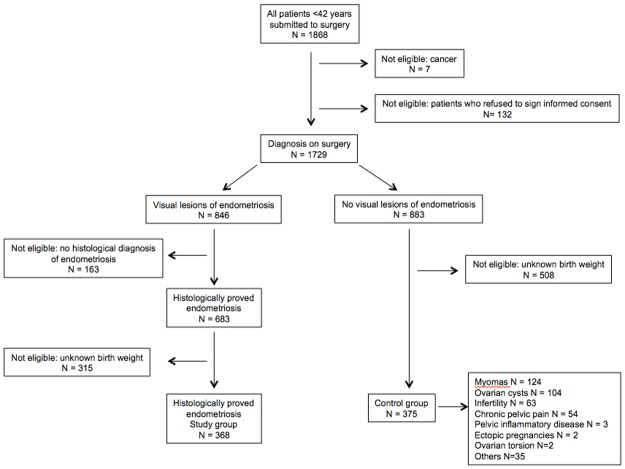
Flow chart of the study.

A flow chart showing the process of patients’ inclusion is presented as Fig. 1. Control group included 375 patients without any visual lesion of endometriosis at the time of surgery. Main indications for surgery in this group, sometimes more than one per patient, were as follows: myomas (124 patients, 33.1%), ovarian cyst (104 patients, 27.7%), infertility (63 patients, 16.8%), chronic pelvic pain (54 patients, 14.4%), pelvic inflammatory disease (3 patients, 0.8%), ectopic pregnancy (2 patients, 0.5%), ovarian torsion (2 patients, 0.5%) and others (35 patients, 9.3%). Study group included 368 patients with histologically proven endometriosis after complete surgical treatment of the lesions. The distribution of patients according to the worst lesion was as follows: SUP (54 patients, 14.7%), OMA (79 patients, 21.5%) and DIE (235 patients, 63.8%). The extent of endometriosis according to the rAFS staging system was classified according to the following groups: stage I: 61 (16.6%) cases; stage II: 80 (21.7%) cases; stage III: 83 (22.6%) cases; and stage IV: 144 (39.1%) cases. Mean rAFS scores were distributed as follows: total: 36.7±32.3, implants: 14.9±12.8 and adhesions: 22.2±25.3. By definition, birth weights were available for all patients both in study and control groups.

Baseline characteristics for cases and controls are presented in Table 1. Significant differences were observed in ethnicity (89.6% patients were Caucasian and 6.3% were Black, in the endometriosis group, vs. 79.7% and 17.6% in the control group, respectively, p<0.001), body mass index, parity, gravidity, oral contraceptives use and infertility (Table 1).

**Table 1:** Patients’ characteristics between control and endometriosis groups

	Endometriosis	Controls	P-value
	N = 368	N = 375	
Age (years) ^a^	31.5 ± 5.4	31.6 ± 6.4	0.93 ^b^
Ethnic group (n, %)			
Caucasian	327 (89.6)	299 (79.7)	
Black	23 (6.3)	66 (17.6)	
Asian	8 (2.2)	2 (0.5)	
Other	7 (1.9)	8 (2.1)	< 0.001 ^c^
Smoking status (n, %)			
Never	190 (51.6)	218 (58.1)	
Former	55 (14.9)	41 (10.9)	
Current	123 (33.4)	113 (30.1)	0.11 ^c^
BMI (kg/m^2^) ^a^	22.1 ± 4.0	22.7 ± 4.3	0.05 ^b^
Parity ^a^	0.26 ± 0.56	0.44 ± 0.89	0.001 ^b^
Gravidity ^a^	0.54 ± 1.00	0.79 ± 1.26	0.003 ^b^
OC use (n, %)			
Never	25 (6.9)	78 (21.0)	
Former	263 (72.2)	199 (53.5)	
Current	76 (20.9)	95 (25.5)	<0.001 ^c^
Infertility (n, %)			
No	239 (65.5)	281 (74.9)	
Primary	100 (27.4)	62 (16.5)	
Secondary	26 (7.1)	32 (8.5)	0.002 ^c^
Infertility as main reason for surgery	40 (10.9)	66 (17.6)	0.009 ^c^
Familial history of endometriosis (n, %)	46 (12.6)	7 (1.9)	<0.001 ^c^
rAFS classification (n, %) ^d^			
I	61 (16.6)		
II	80 (21.7)		
III	83 (22.6)		
IV	144 (39.1)		
rAFS scores^a, d^			
Adhesions	22.2 ± 25.3		
Implants	14.9 ± 12.8		
Total	36.7 ± 32.3		
DIE (n, %) ^e^	235 (63.8)		
USL	52 (22.1)		
Vagina	19 (8.1)		
Bladder	18 (7.7)		
Intestine	128 (54.4)		
Ureter	18 (7.7)		
Number of DIE lesions (n, %)			
1–2	125 (53.2)		
3–4	68 (28.9)		
≥ 5	42 (17.9)		
OMA (n, %)	79 (21.5)		
Right	25 (31.6)		
Left	37 (46.8)		
Bilateral	17 (21.5)		
SUP (n, %)	54 (14.7)		

^a^ Data are presented as mean ± standard deviation;

^b^ Student t-test;

^c^ chi-square test;

^d^ According to the rAFS classification (1985);

^e^ According to a previously published surgical classification for deep endometriosis [32].

OC: oral contraceptive; SUP: superficial endometriosis; OMA: endometrioma; DIE: deep infiltrating endometriosis; USL: utero-sacral ligament.

Mean birth weights according to the indication for surgery in control group were the following: myomas: 3,259g; ovarian cyst: 3,256g; infertility: 3,265g; chronic pelvic pain: 3,246g; emergency (including ectopic pregnancy, tubal infection and ovarian torsion): 3,660g and others: 3,307g. There were no significant differences of birth weight according to the indications for surgery in the control group (p-value = 0.57, chi-square test, patients with multiple indications were excluded from the statistical analysis). Mean birth weight was significantly lower for patients with endometriosis as compared to controls (3,119g ± 614 and 3,251g ± 557 respectively; p = 0.002) (Table 2) and the OR for each 100g more is: OR = 0.96 (95%CI: 0.94–0.99, p = 0.002). Eleven patients with endometriosis (3.0%) had a birth weight below 1,500g whereas all women in the control group had a birth weight above 1,500g (Table 2). Mean birth weight was also significantly different among the endometriosis groups according to the surgical classification (p-value = 0.02 for ANOVA between controls, SUP, OMA, and DIE, Table 2). When comparing SUP to controls and OMA to controls, the quantitative difference in birth weight did not reach significance: 3,110 g ± 597 and 3,172 g ± 611 vs. 3,251g ± 557 respectively; p = 0.09 for SUP and p = 0.26 for OMA), but two-by-two comparisons with controls reached significance for all types of endometriosis when birth weight was considered in classes (Table 2). Conversely, there were no differences of birth weight according to the laterality of OMA (left, right or bilateral: 3,228g ± 704, 3,116g ± 539 and 3,133g ± 507 respectively; p = 0.20).

**Table 2:** Distribution of women with endometriosis and controls according to birth weight categories.

	Controls	Endometriosis	P-value	SUP	P-value	OMA	P-value	DIE	P-value	Global P-value
	N = 375	N = 368		N = 54		N = 79		N = 235		
Mean birth weight ^a^	3,251 ± 557	3,119 ± 614	0.002 ^b^	3,110 ± 597	0.09 ^b^	3,172 ± 611	0.26 ^b^	3,103 ± 620	0.002 ^b^	0.02 ^c^
Birth weight (n, %)										
≤ 1,500g	0 (0.0)	11 (3.0)		2 (3.7)		2 (2.5)		7 (3.0)		
1,501g—2,500g	37 (9.9)	42 (11.4)		4 (7.4)		8 (10.1)		30 (12.7)		
2,501g—4,000g	316 (84.3)	304 (82.6)		48 (88.9)		67 (84.8)		189 (80.4)		
> 4,000g	22 (5.9)	11 (3.0)	0.002 ^d^	0	0.006 ^e^	2 (2.5)	0.049 ^e^	9 (3.8)	0.003 ^d^	0.03 ^f^

^a^ Data are presented as mean ± standard deviation;

^b^ Student t-test for two-by-two comparison to controls;

^c^ ANOVA comparing all 4 groups (controls, SUP, OMA and DIE)

^d^ Chi-square test;

^e^ Fischer exact test;

^f^ Fischer exact test comparing all 4 groups of endometriosis (controls, SUP, OMA and DIE), according to birth weight category;

SUP: superficial endometriosis; OMA: endometrioma; DIE: deep infiltrating endometriosis

When compared to controls, patients with DIE had the lowest birth weight with a highly significant difference (3,103g ± 620 vs. 3,251g ± 557, p = 0.002). Among patients with DIE, there were no differences in birth weight according to the number of deep lesions (one lesion: 3,135g ± 665, two lesions: 3,156g ± 636, three and four lesions: 3,154g ± 569 and more than five: 3,134g ± 612; p = 0.64) and the main localization (utero-sacral ligaments and vagina: 3,099g ± 739; bladder, intestine and ureter: 3,100g ± 560; p = 0.99). As well, there were no differences of birth weight according to the rAFS scoring system.

Results of logistic regression analysis are shown in Table 3. In univariate analysis, patients with a birth weight below 2,500g had a higher risk of endometriosis, especially DIE, as compared to the reference group (OR = 1.5, 95%CI: 1.0–2.3 and OR = 1.7, 95%CI: 1.0–2.7, respectively). Multivariate analysis, adjusted on ethnicity and smoking status, showed the persistence of a significant association between endometriosis and the group with a birth weight below 2,500g, with a slight increase in the magnitude of the association (aOR = 1.7, 95%CI: 1.0–2.6). Patients with a birth weight below 2,500g had also a higher risk of DIE (aOR = 1.8; 95%CI: 1.1–2.9). There was no significant increase in risk for SUP or OMA in this group of patients with a LBW. Finally, we observed that in the group of women with birth weight > 4,000g, OR and aOR for endometriosis, for DIE or for OMA were persistently < 1, and mostly ≤ 0.5, suggesting a dose-effect relation between birth weight and endometriosis, although it did not reach significance in any of the three groups.

**Table 3:** Association between birth weight and endometriosis.

Birth weight (g)	≤ 2,500	2,501–4,000	> 4,000
All endometriosis (N = 368)			
N (%)	53 (14.4)	304 (82.6)	11 (3.0)
OR (95%CI)	1.49 (0.95–2.33)	Reference	0.52 (0.25–1.09)
aOR (95%CI)	1.65 (1.04–2.62)	Reference	0.49 (0.23–1.05)
SUP (N = 54)			
N (%)	6 (11.1)	48 (88.9)	0
OR (95%CI)	1.07 (0.43–2.66)	Reference	NA
aOR (95%CI)	1.23 (0.47–3.21)	Reference	NA
OMA (N = 79)			
N (%)	10 (12.7)	67 (84.8)	2 (2.5)
OR (95%CI)	1.27 (0.60–2.69)	Reference	0.43 (0.10–1.87)
aOR (95%CI)	1.52 (0.70–3.31)	Reference	0.47 (0.10–1.89)
DIE (N = 235)			
N (%)	37 (15.8)	189 (80.4)	9 (3.8)
OR (95%CI)	1.67 (1.02–2.73)	Reference	0.68 (0.31–1.52)
aOR (95%CI)	1.78 (1.08–2.94)	Reference	0.63 (0.28–1.41)

OR: odds ratio;

aOR: adjusted odds ratio; 95%CI: 95% confidence interval;

SUP: superficial endometriosis; OMA: endometrioma; DIE: deep infiltrating endometriosis;

Multivariate analysis has been adjusted for ethnicity and smoking status. Women with a birth weight between 2,501g and 4,000g served as a reference population.

NA: not applicable, no patient in this category.

## Discussion

After adjustment for smoking and ethnicity (two factors that possibly influence birth weight), we observed that women with surgically confirmed and histologically staged endometriosis had a lower birth weight than controls in which the disease was surgically excluded with an absolute certitude. We found a significant association between DIE and LBW (i.e. weight at term delivery below 2,500g) while the difference did not reach significance for SUP and OMA. To the best of our knowledge, this is the first report highlighting a strong association between LBW and DIE. This result is important as it may help, together with known risk factors (low BMI, NSAID-resistant dysmenorrhea, OC pill) to discriminate the patients with a higher risk of severe endometriosis (DIE) and to chose the appropriate imaging work-up.

Our findings are in line with previous reports documenting a positive association between LBW and endometriosis. In a cohort study of 1,226 cases of laparoscopically-confirmed endometriosis, Missmer et al. reported an increased risk of endometriosis for LBW with a relative risk of 1.3 (95%CI: 1.0–1.8, p < 0.01) [7]. By contrast, Somigliana et al. failed to detect any association between endometriosis and birth weight [8]. These authors recruited 91 women with a laparoscopic diagnosis of endometriosis and 82 controls who underwent operative laparoscopy and were free of disease in a case-control study design. It is likely that the small number of patients enrolled in the study accounted for the negative results, while the strongest association between endometriosis and birth weight was observed in DIE, which afflicted most of our patients. The ENDO study reported recently that maternal behaviors during pregnancy and LBW in particular did not significantly increase the risk of endometriosis (aOR = 1.1; 95%CI: 0.92–1.32) [9]. However, in this cohort study specifically designed to assess in utero exposures and endometriosis risk, the proportion of histologically proven endometriosis was very small (68 patients) and the disease was not categorized into groups such as SUP, OMA and DIE. Therefore, negative conclusions of the study are certainly not definitive and do not apply to specific groups of endometriosis, such as DIE which was the most common group in our population.

Somigliana et al. have nicely depicted the limitations of such observational studies whose negative effects could be amplified by the long time gap between prenatal exposure and outcome in adulthood [8]. In our study, assessment of birth weight based on medical records made our results presumably less exposed to recall bias. While we concede easily with Somigliana that there is no ideal control group for epidemiologic studies on endometriosis, we have considered that excluding endometriosis at the time of surgery was the best way to undoubtedly select healthy controls. Concerning the study group misdiagnoses were highly improbable because all cases underwent complete excision of all visible endometriotic lesion followed by a systematic pathologic confirmation. More than 50% of our patients in the study group had either stage III or IV, or DIE. This reflected obviously our referral pattern. And concerning the control group, although they may differ from the endometriosis group in many ways, none of the conditions affecting the control group was ever reported to be influenced by birth weight. Consequently, the association found should not be biased by the selection of patients. In this way our selection criteria for recruiting patients and controls were even more stringent than those proposed by Holt and Weiss [17].

A limitation to the interpretation of our results is due to the fact that the medical charts we consulted did not mention the gestational age at delivery, which could obviously influence the birth weight. In the ENDO study and other studies, preterm delivery was not a risk factor for endometriosis (OR = 0.98; 95%CI 0.47–2.03) [8,9]. On the contrary, the authors showed that preterm birth significantly decreased the odds of disease in analyses restricted to visually and histologically confirmed endometriosis (aOR = 0.41; 95%CI: 0.18–0.94). In other words, it is probable that the association observed between LBW and endometriosis is due more to intrauterine growth restriction than to prematurity.

The influence of intrauterine environment on the risk of endometriosis is still controversial [18]. In this context the rationale for the association with LBW is unknown. In a mice model submitted to maternal utero-placental insufficiency, restricted female offspring exhibited growth restriction associated with biologic abnormalities including elevated triglycerides, uterine endothelial dysfunction and increased uterine artery rigidity [19]. Decreased elasticity of the pelvic vessels and pathologic development of connective tissue associated with LBW may hamper menstrual flow throughout the cervix [20]. This may increase in return the volume of retrograde menstruation favoring the development of endometriotic lesions [16,21,22]. Another hypothesis incriminates the insulin and insulin-like growth factors (IGF) pathways. Data showed that LBW and intrauterine growth restriction could affect the insulin and IGF axes around birth [23]. In endometriotic lesions insulin and IGF pathways are altered as compared to normal endometrium [24,25]. A recent DNA methylation and transcription profiling identified 23 genes whose methylation levels explained 70–87% of the variance in birth weight [26]. These genes are involved in oxidative stress and insulin signaling, both pathways strongly modified during endometriosis development [24,27].

The hypothesis has been formulated that neonatal uterine bleeding and endometrial stem/progenitor cells may play a critical role in the development of early-onset endometriosis and explain the severity of endometriosis in the adolescent [28,29]. Withdrawal from the maternal environment of high estrogen and progestogen concentrations at full-term birth may affect the secretory neonatal endometrium by producing menstruation-like changes. Uterine bleeding has been documented to occur from around day 3 for a period of 3 to 5 days and is overt in 3–6% and occult in 25–50% [30]. As stated above, prematurity seems to decrease the risk of endometriosis in adulthood [9]. This finding would be in agreement with the likely absence of neonatal uterine bleeding in preterm neonates when no secretory changes have occurred. In contrast, in full-term pregnancies, the neonatal uterine structure with a cervical canal twice the length of the corpus with thick mucus may favor retrograde menstruation. This may be even more pronounced if utero-placental insufficiency, reflected by LBW, has hampered the normal development of pelvic vessels and connective tissue, explaining why LBW seems to positively influence the development of severe endometriosis. It is important to note that endometrial changes in 170 newborns as described by Ober and Bernstein (1955) included four cases with decidual and five cases with menstrual changes. The reported birth weight for the first group varied between 1,600g and 2,950g and the second group between 2,400g and 3,280g [31].

To conclude, this 743 case-control study provides evidence for an association between LBW and endometriosis, and especially DIE. While this association could reflect common signaling pathways between endometriosis and fetal growth regulation, there is also the possibility of a role played by neonatal uterine bleeding in the risk of severe endometriosis.

## Supporting Information

S1 STROBE Checklist (PDF)
